# Reduced prefrontal hemodynamic response in pediatric autism spectrum disorder measured with near-infrared spectroscopy

**DOI:** 10.1186/s13034-019-0289-9

**Published:** 2019-06-28

**Authors:** Mitsuhiro Uratani, Toyosaku Ota, Junzo Iida, Kosuke Okazaki, Kazuhiko Yamamuro, Yoko Nakanishi, Naoko Kishimoto, Toshifumi Kishimoto

**Affiliations:** 1 Department of Psychiatry, Manyo Hospital, Kashihara, Japan; 2 Department of Psychiatry, Nara Medical University, 840 Shijyo-cho, Kashihara, Nara 634-8522 Japan; 3 Faculty of Nursing, Nara Medical University, Kashihara, Japan

**Keywords:** Pediatric autism spectrum disorder, Near-infrared spectroscopy, Prefrontal hemodynamic response, Attention, Executive function

## Abstract

**Background:**

Functional neuroimaging studies suggest that prefrontal cortex dysfunction is present in people with autism spectrum disorder (ASD). Near-infrared spectroscopy is a noninvasive optical tool for examining oxygenation and hemodynamic changes in the cerebral cortex by measuring changes in oxygenated hemoglobin.

**Methods:**

Twelve drug-naïve male participants, aged 7–15 years and diagnosed with ASD according to DSM-5 criteria, and 12 age- and intelligence quotient (IQ)-matched healthy control males participated in the present study after giving informed consent. Relative concentrations of oxyhemoglobin were measured with frontal probes every 0.1 s during the Stroop color-word task, using 24-channel near-infrared spectroscopy.

**Results:**

Oxyhemoglobin changes during the Stroop color-word task in the ASD group were significantly smaller than those in the control group at channels 12 and 13, located over the dorsolateral prefrontal cortex (FDR-corrected P: 0.0021–0.0063).

**Conclusion:**

The results suggest that male children with ASD have reduced prefrontal hemodynamic responses, measured with near-infrared spectroscopy.

## Background

Autism spectrum disorder (ASD) is a neurodevelopmental disorder, characterized by impairments in social and communicative functioning and the presence of restricted interests and repetitive behaviors [1]. Studies using neuropsychological measures have revealed an association between ASD and inattention. ASD can be characterized by a short attention span, and impulsivity and inattention symptoms are common [2]. Furthermore, individuals with ASD are typically impaired on neurocognitive measures of sustained and selective attention [3]. There is evidence for fronto-striatal, parietal, and cerebellar abnormalities in ASD during selective and flexible attention [4, 5]. In addition to attentional difficulties, many studies have indicated that individuals with ASD exhibit impairments of executive function [6, 7]. A wealth of data indicates that the prefrontal cortex plays a major role in executive function.

Multi-channel near-infrared spectroscopy (NIRS) enables the noninvasive detection of neural activity near the surface of the brain using near-infrared light [8, 9]. NIRS measures alterations in oxygenated hemoglobin (oxy-Hb) and deoxygenated hemoglobin (deoxy-Hb) concentrations in micro-blood vessels on the brain surface. Local increases in the concentration of oxy-Hb and decreases in the concentration of deoxy-Hb are indicators of cortical activity [8, 10]. In addition, changes in the concentration of oxy-Hb have been associated with changes in regional cerebral blood volume, using a combination of positron emission tomography (PET) and NIRS measurements [11, 12]. NIRS is a neuroimaging modality that, according to Matsuo et al. [13], is especially suitable for psychiatric patients for the following reasons. First, because NIRS is relatively insensitive to motion artifacts, it can be used in experiments where motion is expected, such as those involving vocalization. Second, NIRS can be used to examine participants while seated in a natural position, with minimal environmental distraction. Third, NIRS has cheaper running costs than other neuroimaging modalities and is simple to set up and use. Fourth, the high temporal resolution of NIRS is useful for characterizing the time course of prefrontal activity in people with psychiatric disorders [14, 15]. Accordingly, NIRS has been used to assess brain function in people with many psychiatric disorders, including schizophrenia, bipolar disorder, depression, obsessive–compulsive disorder, dementia, post traumatic stress disorder, Tourette’s disorder, attention-deficit/hyperactivity disorder, and ASD [13–27].

Recent developments in NIRS have enabled noninvasive clarification of brain functions in pediatric psychiatric disorders. In pediatric ASD, reduced prefrontal hemodynamic activity has been reported in studies using NIRS measurement during self-face recognition and auditory tasks [28, 29]. Yasumura et al. [30] reported no significant differences in prefrontal hemodynamic activity between typically developing and ASD children (seven boys and four girls) measured with NIRS during the Stroop task. Similarly, Xiao et al. [31] reported no significant differences in prefrontal hemodynamic activity between typically developing controls and boys with ASD measured with 16-channel NIRS during the Stroop task. However, it is difficult to accurately measure the dorsolateral prefrontal hemodynamic activity using 16-channel NIRS, which is more suitable for measuring hemodynamic responses of the orbitofrontal and frontopolar cortex. The Stroop color-word task is one of the most commonly used methods for identifying attentional problems, as well as providing a test of executive function, and involves the dorsolateral prefrontal cortex. Moreover, sex differences in executive function in people with ASD have been reported in children and adolescents [32–34]. Therefore, it may be valuable to examine the broader prefrontal hemodynamic response in male children with ASD, measured with 24-channel NIRS during the Stroop color-word task. We hypothesized that male children with ASD would exhibit reduced prefrontal hemodynamic responses in 24-channel NIRS during the Stroop color-word task. Thus, in the present study, we used 24-channel NIRS to examine the characteristics of prefrontal cerebral blood volume changes during the Stroop color-word task in male children with ASD and in age- and intelligence quotient (IQ)-matched healthy control males.

## Methods

### Participants

Twelve drug-naïve male participants, aged 7–15 years, and diagnosed with ASD according to DSM-5 criteria [1], were compared with 12 age- and IQ-matched healthy control males, aged 6–12 years (Table 1).

**Table 1:** Participants’ characteristics

	ASD	Control	*P* value
	Mean (SD)	Mean (SD)	
Number (sex ratio: M:F)	12 (12:0)	12 (12:0)	
Age (years)	9.75 (2.26)	9.50 (2.20)	0.79
First diagnosed age (years)	8.17 (1.95)	NA	
FIQ (WISC-IV)	100.92 (15.72)	97.83 (7.66)	0.55
SCWC-1	34.58 (12.32)	38.58 (7.13)	0.34
SCWC-2	36.92 (10.47)	38.58 (7.96)	0.67
SCWC-3	35.42 (11.98)	37.08 (9.10)	0.71

Group differences tested with *t*-test

*ASD* autism spectrum disorder, *M* male, *F* female, *FIQ* (WISC-IV) Full-scale IQ score of the Wechsler Intelligence Scale for Children-Fourth Edition, *SCWC-1* Stroop color-word task number of correct answers first time, *SCWC-2* Stroop color-word task number of correct answers second time, *SCWC-3* Stroop color-word task number of correct answers third time

Participants were individuals with ASD who had no history of previous psychiatric disorder treatment, and had consulted one of the experienced pediatric psychiatrists at the Department of Psychiatry of Nara Medical University that anyone with demand could visit at any time without constraints of severity, age, residence, economics, and so on. Participants with ASD underwent a standard clinical assessment comprising a psychiatric evaluation, a semi-structured interview system for ASD (the Pervasive Developmental Disorders Assessment System) [35], and an examination of medical history by an experienced pediatric psychiatrist. Two experienced pediatric psychiatrists confirmed the diagnosis of ASD in accordance with the DSM-5. Participants’ intellectual level was assessed using the Wechsler Intelligence Scale for Children–Fourth Edition by the psychologist, and individuals with full-scale IQ (FIQ) scores below 70 were excluded. Patients who presented with a comorbid psychiatric disorder defined by the DSM-5, a neurological disorder, a head injury, a serious medical condition, or a history of substance abuse/dependence were excluded; two patients with attention-deficit/hyperactivity disorder and two patients with persistent motor tic disorder were excluded. Finally, 12 participants with ASD, who had no previous medication history, were enrolled in the present study. Of 12 participants, two had been previously diagnosed by the pediatric neurologist at the other hospital, three had been previously diagnosed by using the Autism Diagnostic Interview Revised, one had been previously diagnosed by using the Autism Diagnostic Observation Schedule, and other participants were diagnosed for the first time at the Department of Psychiatry of Nara Medical University.

Healthy control participants were recruited from local elementary schools and junior high schools. They also underwent a standard clinical assessment comprising a psychiatric evaluation, a standard diagnostic interview (Structured Clinical Interview for DSM-IV-TR Axis I Disorders Non-Patient Edition), and an examination of medical history by an experienced pediatric psychiatrist. Participants’ intellectual level was assessed using the Wechsler Intelligence Scale for Children-Fourth Edition by the psychologist. Finally, 12 healthy control participants, who did not have confirmed ASD and who had no current or past history of psychiatric or neurological disorders, were also enrolled in the present study.

All participants were able to read the Japanese syllabary characters called hiragana, right-handed and Japanese. All participants and/or their parents provided written informed consent for their participation in the study. We informed our patients about this study on their initial visit and enrolled them as the participant of this study in order of consent. This study was approved by the Institutional Review Board at the Nara Medical University (approval number 325-2).

### The Stroop color-word task

The traditional Stroop task involves a word-reading task, an incongruent color naming task, and a color naming task. We reconstructed the Stroop task according to previously described methods [36]. The Stroop color-word task consisted of two pages: each page contained 100 items in five columns of 20 items each and the page size was 210 × 297 mm. On the first page, the words RED, GREEN, and BLUE were printed in black ink. On the second page, the words RED, GREEN, and BLUE were printed in red, green, or blue ink, with the limitation that the word meaning and ink color never matched. The items on both pages were randomly distributed, with the exception that no item could appear directly after the same item within a column.

Before the task, the examiners gave the participants the following instructions: “This task is to test how quickly you can read the words on the first page, and say the colors of the words on the second page. After we say ‘begin’, please read the words in the columns, starting at the top left, and say the words/colors as quickly as you can. After you finish reading the words in the first column, go on to the next column, and so on. After you have read the words on the first page for 45 s, we will turn the page. Please repeat this procedure for the second page.”

**Fig. 1:**
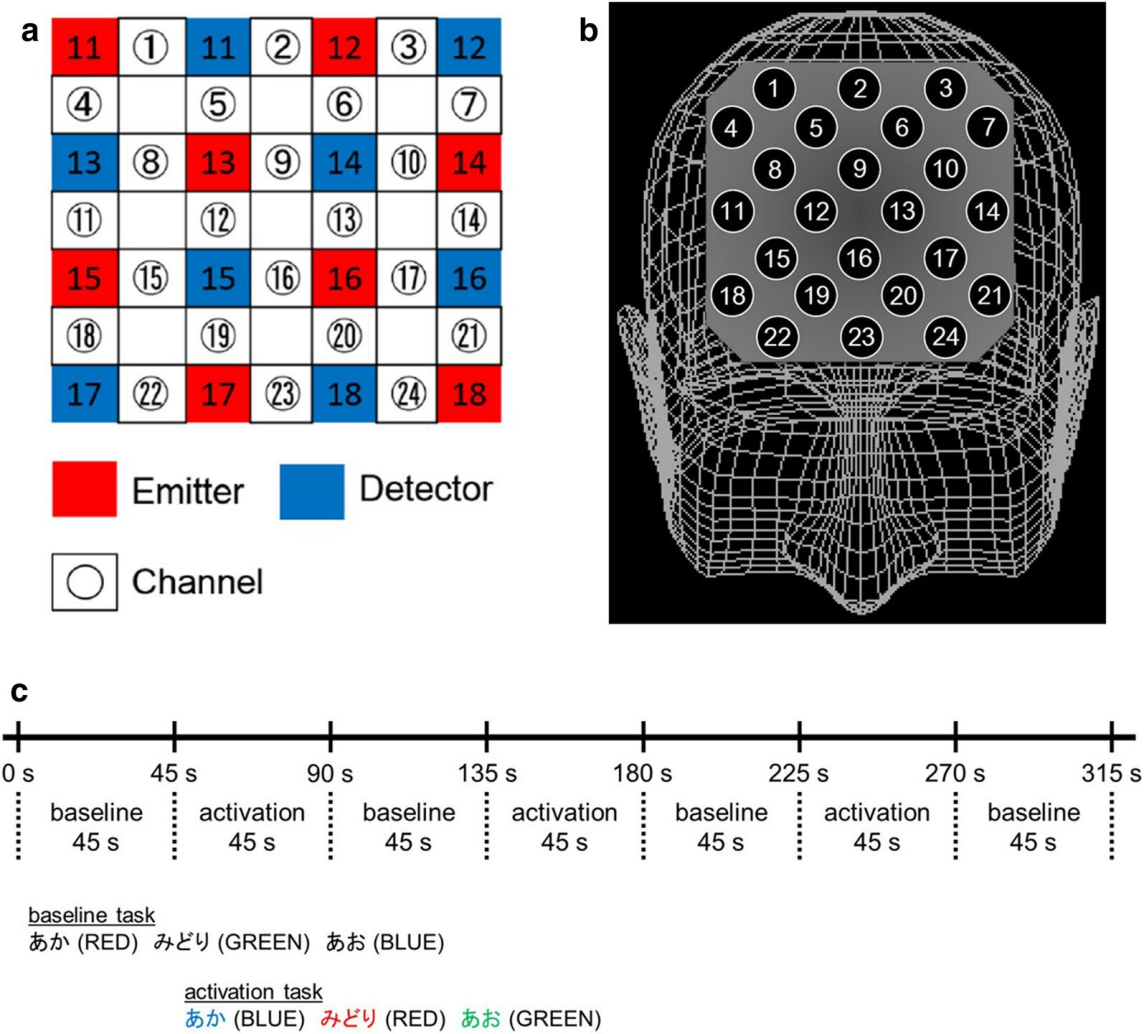
Location of the 24 channels of the near-infrared spectroscopy device. **a** Arrangement of emitters and detectors according to the definition of each channel. **b** Corresponding anatomical site of each channel. **c** Timeline of stimulus presentation. The baseline task is the word reading task. The activation condition is the incongruent color naming task

The entire Stroop color-word task sequence consisted of three cycles of 45-s spent reading the first page, 45-s spent reading the second page (the color-word task). The task ended with 45-s spent reading the first page, which we designated as the baseline task (Fig. 1c). We recorded the number of correct answers in each cycle, and refer to them as follows: Stroop color-word task number of correct answers first time (SCWC-1), second time (SCWC-2), and third time (SCWC-3). Examiners who were blind to the participants’ diagnoses administered the Stroop color-word task.

Importantly, the Stroop task used in this study was different to the traditional Stroop task. We used a simplified version of the Stroop color-word task because the participants were school-age children. In addition, we excluded the color-naming task (part of the traditional Stroop task) because we needed only two tasks (baseline task and activation task) for our NIRS study.

The Stroop color-word task was utilized for the following reasons. First, the inferior frontal gyrus is reported to be one of the regions most strongly related to Stroop interference [37]. Second, in the NIRS study that the same task was used, Negoro et al. [26] concluded that suitable prefrontal brain activation in healthy children was recognized by using the Stroop color-word task.

### NIRS measurements

Increased oxy-Hb and decreased deoxy-Hb, measured with NIRS, have been reported to reflect cortical activation. In animal studies, oxy-Hb is the most sensitive indicator of regional cerebral blood flow because the direction of change in deoxy-Hb is determined by the degree of change in venous blood oxygenation and volume [38]. Therefore, we focused on changes in oxy-Hb. We measured oxy-Hb using a 24-channel NIRS machine (Hitachi ETG-4000, Hitachi Medical Corporation, Tokyo, Japan). We measured the absorption of two wavelengths of near-infrared light (760 and 840 nm). We analyzed the optical data based on the modified Beer–Lambert Law [39] as previously described [40]. This method enabled us to calculate signals reflecting oxy-Hb, deoxy-Hb, and total-Hb signal changes. The scale of the hemoglobin quantity is mmol × mm, meaning that all concentration changes depend on the path length of the near-infrared light. The recording channels were located over the optical path in the brain between neighboring pairs of emitters and detectors (Fig. 1a). The inter-probe intervals of the system were 3.0 cm, and previous reports have established that the device measures activity at a point 2–3 cm beneath the scalp (i.e., the surface of the cerebral cortex) [19, 41].

The participants maintained a natural sitting position during NIRS measurements. The distance between the eyes of each participant and the paper on which the items were listed was set to between 30 cm and 40 cm. The NIRS probes were placed on the scalp over the prefrontal brain regions, and arranged to measure the relative changes in Hb concentration at 24 measurement points that made up an 8 × 8 cm square (Fig. 1a). The lowest probes were positioned along the Fp1–Fp2 line, according to the international 10/20 system commonly used in electroencephalography. The probe positions and measurement points on the cerebral cortex were confirmed by overlaying the probe positions on a three-dimensionally reconstructed magnetic resonance imaging scan of the cerebral cortex of a representative participant from the control group (Fig. 1b). The absorption of near-infrared light was measured with a time resolution of 0.1 s. The data were analyzed using the “integral mode”: the pre-task line was determined as the mean across the 10 s just before the task period; the post-task line was determined as the mean across the 25 s immediately after the task period; using two lines, the baseline was drawn using the least-squares method; the three oxy-Hb changes of the activation task were then averaged. The moving average method was used to exclude short-term motion artifacts in the analyzed data (moving average window, 5 s).

We attempted to exclude motion artifacts by closely monitoring artifact-evoking body movements, such as neck movements, biting, and blinking (identified as the most influential in a preliminary artifact-evoking study), and by instructing the participants to avoid these movements during the NIRS measurements. Examiners were blind to the participants’ diagnoses.

### Statistical analyses

**Fig. 2:**
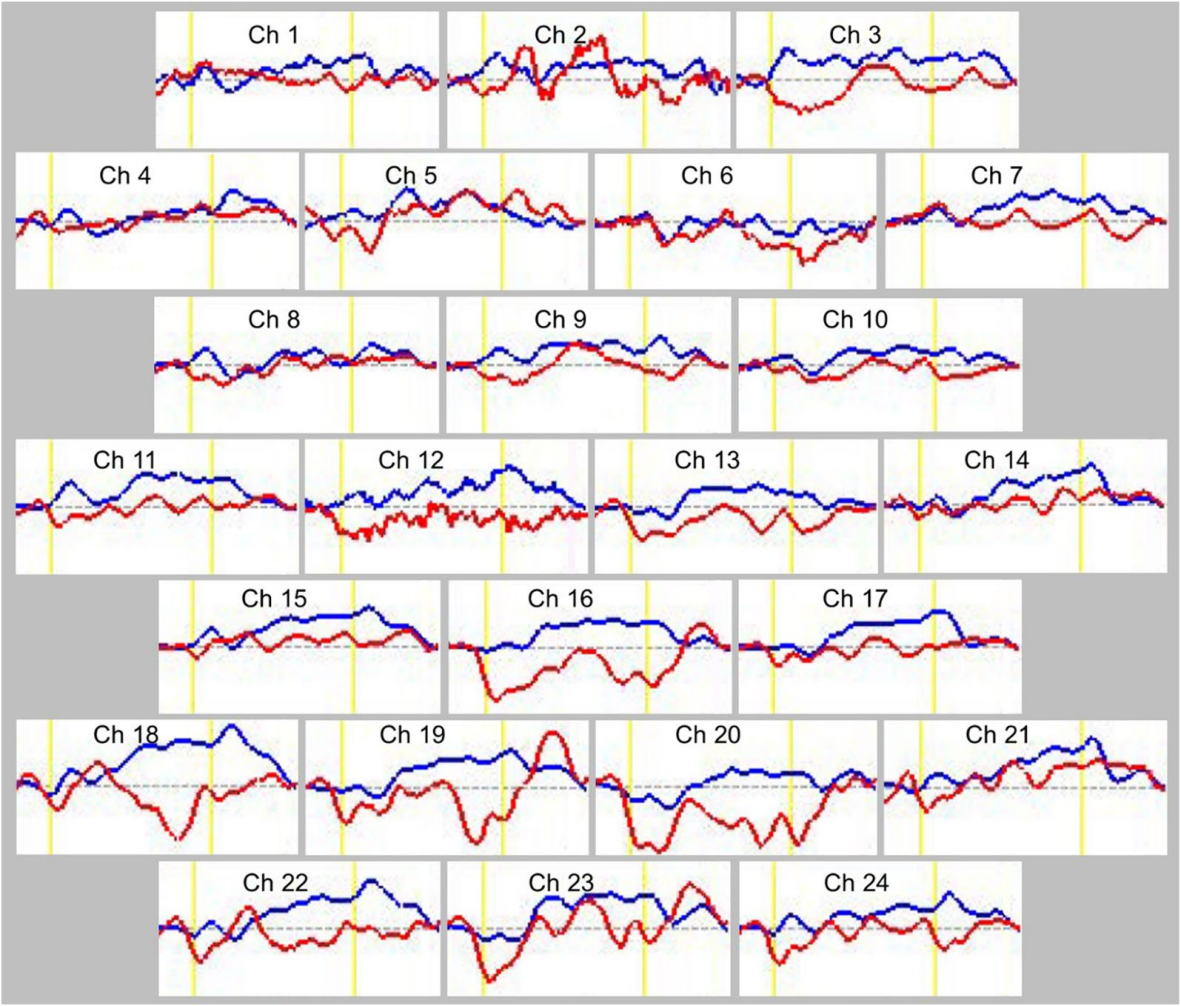
Grand average waveforms of oxyhemoglobin (oxy-Hb) concentration changes during the Stroop color-word task in both groups. The red lines are the grand average waveforms of oxy-Hb in the autism spectrum disorder (ASD) group, and the blue lines are the grand average waveforms of oxy-Hb in the control group. The activation task was performed in the time period between the yellow lines

We used Student’s *t*-tests to compare oxy-Hb changes between the two groups by calculating the grand average waveforms every 0.1 s in each channel. This analysis enabled more detailed comparison of oxy-Hb changes along the time course of the task. Data analyses were conducted using MATLAB 6.5.2 (Mathworks, Natick, MA, USA) and Topo Signal Processing type-G version 2.05 (Hitachi Medical Corporation, Tokyo, Japan). OT-A4 version 1.63 K (Hitachi Medical Corporation, Tokyo, Japan) was used for the overlap display of the grand average waveforms in both groups in Fig. 2 and was also used to calculate mean oxy-Hb measurements in Table 2. Because we performed 24 paired *t*-tests, correction for multiple comparisons was conducted using the false discovery rate (FDR) (two-tailed; we set the value of *q* specifying the maximum FDR to 0.05, so that there are no more than 5% false positives on average) [42]. PASW Statistics 18.0 J for Windows (SPSS, Tokyo, Japan) was used for statistical analysis.

**Table 2:** Correlations between Stroop task and participants’ characteristics

	ASD			Control		
	SCWC-1	SCWC-2	SCWC-3	SCWC-1	SCWC-2	SCWC-3
Age	0.790*	0.582*	0.618*	0.577*	0.576*	0.598*
FIQ (WISC-IV)	− 0.165	− 0.187	− 0.240	− 0.272	− 0.279	− 0.290

Correlations between Stroop task and participants’ characteristics tested with Spearman’s correlation test

*ASD* autism spectrum disorder, *FIQ (WISC-IV)* Full-scale IQ score of the Wechsler Intelligence Scale for Children-Fourth Edition, *SCWC-1* Stroop color-word task number of correct answers first time, *SCWC-2* Stroop color-word task number of correct answers second time, *SCWC-3* Stroop color-word task number of correct answers third time

* P < 0.05

** P < 0.01

## Results

### Demographic data

Demographic and clinical data are shown in Table 1. Age and FIQ did not differ significantly across patients with ASD and healthy controls (t = 0.28, *df* = 22, P = 0.79; t = 0.61, *df* = 22, P = 0.55). There were no significant differences in the SCWC-1, SCWC-2, and SCWC-3 scores between the two groups (t = − 0.97, *df* = 22, P = 0.34; t = − 0.44, *df* = 22, P = 0.67; t = − 0.38, *df* = 22, P = 0.71).

### Correlation between the Stroop task and characteristics of the participants

Spearman’s ρ correlations between SCWC scores and age, and FIQ scores are shown in Table 2. In both groups, the results revealed positive correlations between SCWC scores and age, and no correlations between SCWC scores and FIQ.

### NIRS data during the stroop color-word task

**Fig. 3:**
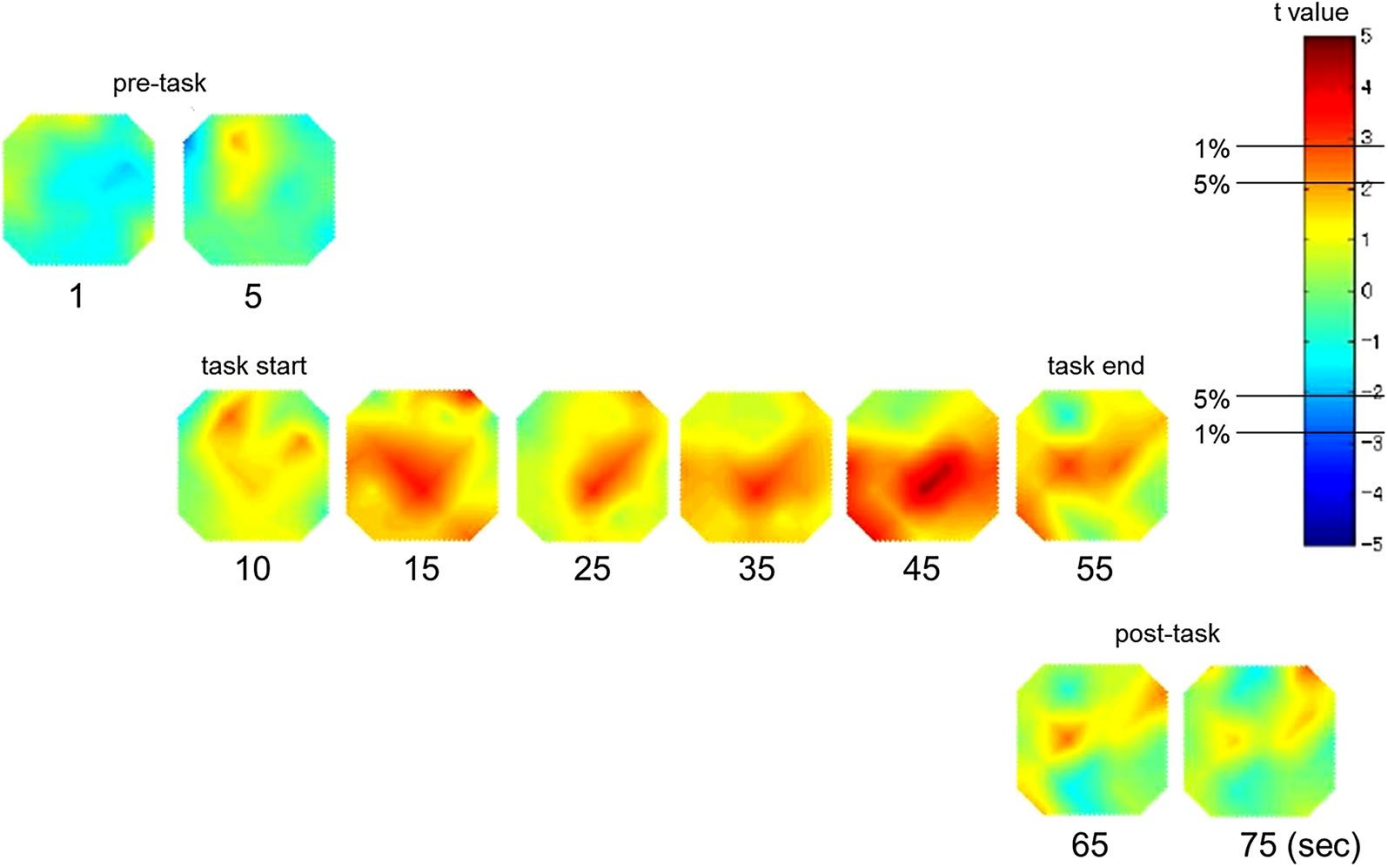
Topographic presentation of the *t* value of the oxyhemoglobin (oxy-Hb) comparison between the control group and the autism spectrum disorder (ASD) group during the Stroop color-word task. The *t* values of oxy-Hb for the control and ASD groups are presented as a topographic map along the time course of the task (from top to bottom). The red, green, and blue areas in the topographs indicate positive, zero, and negative *t* values, with ± 2.8 and ± 2.1 for the 1% and 5% statistical significance levels, respectively

The grand average waveforms of oxy-Hb concentration changes during the Stroop color-word task in both groups can be seen in Fig. 2. The grand average waveforms of the oxy-Hb concentration change in the control group increased during the task period, whereas those in the ASD group did not show substantial changes. The difference in mean oxy-Hb measurements between the task and post-task periods in 24-channels NIRS is shown in Table 3. Between the task and post-task periods, the mean oxy-Hb difference of the ASD group was significantly smaller than that of the control group in channels 12 and 13 (FDR-corrected P: 0.0021 to 0.0042). A topographic representation of the t-values of oxy-Hb comparison between the ASD group and the control group during the Stroop color-word task is shown in Fig. 3. The oxy-Hb changes in the control group were significantly greater than with those in the ASD group during the task period in the prefrontal cortex.

**Table 3:** Difference of mean oxyhemoglobin (oxy-Hb) measurements between task and post-task periods in 24 channels

	ASD (mMmm)		Control (mMmm)		Student’s t-test	FDR correction
	Mean	SD	Mean	SD		
Ch 1	0.0049	0.0658	0.0210	0.0447	NS	NS
Ch 2	0.0055	0.0511	0.0267	0.0653	NS	NS
Ch 3	− 0.0161	0.0987	0.0427	0.0444	†	NS
Ch 4	0.0085	0.0470	0.0146	0.0423	NS	NS
Ch 5	0.0203	0.0728	0.0239	0.0414	NS	NS
Ch 6	− 0.0323	0.0586	− 0.0087	0.0329	NS	NS
Ch 7	− 0.0003	0.0483	0.0301	0.0454	NS	NS
Ch 8	− 0.0014	0.0560	0.0257	0.0498	NS	NS
Ch 9	− 0.0016	0.0515	0.0312	0.0307	†	NS
Ch 10	− 0.0121	0.0576	0.0288	0.0451	†	NS
Ch 11	− 0.0005	0.0489	0.0349	0.0345	†	NS
Ch 12	− 0.0299	0.0567	0.0396	0.0358	**	***
Ch 13	− 0.0317	0.0483	0.0255	0.0352	**	***
Ch 14	0.0058	0.0814	0.0334	0.0167	NS	NS
Ch 15	0.0134	0.0705	0.0438	0.0271	NS	NS
Ch 16	− 0.0420	0.0469	0.0306	0.0198	NS	NS
Ch 17	− 0.0037	0.0680	0.0298	0.0188	NS	NS
Ch 18	− 0.0189	0.0712	0.0651	0.0462	**	NS
Ch 19	− 0.0145	0.1062	0.0327	0.0460	NS	NS
Ch 20	− 0.0718	0.1045	0.0005	0.0471	*	NS
Ch 21	0.0239	0.0805	0.0480	0.0318	NS	NS
Ch 22	− 0.0144	0.0544	0.0328	0.0578	†	NS
Ch 23	0.0028	0.1099	0.0381	0.0507	NS	NS
Ch 24	− 0.0106	0.0858	0.0318	0.0345	NS	NS

Group differences tested with *t*-test and false discovery rate (FDR) correction

* P < 0.05

** P < 0.01

*** P < FDR-corrected P

^†^P < 0.1

## Discussion

To the best of our knowledge, no previous studies have examined the broader prefrontal hemodynamic response in male children with ASD, measured with 24-channel NIRS during the Stroop color-word task. In the present study, the results revealed that oxy-Hb changes in 12 drug-naïve male children with ASD during the Stroop color-word task were significantly smaller than those in 12 healthy male children in the prefrontal cortex, particularly in the dorsolateral prefrontal cortex (Ch 12 and Ch 13). The present findings supported our hypothesis, in accord with the proposed prefrontal dysfunction in pediatric ASD identified by other imaging modalities, such as functional magnetic resonance imaging (fMRI) and single-photon emission computed tomography (SPECT). Previous SPECT studies reported localized areas of hypoperfusion, which may be correlated with focal reductions in function observed in the prefrontal lobes, cingulate gyrus, superior temporal gyrus, and mesial temporal lobes of individuals with ASD [43]. In ASD, fMRI studies of motor and cognitive interference inhibition and switching reported abnormalities in fronto-striato-parietal areas, including dorsolateral prefrontal cortex and ventrolateral prefrontal cortex [44, 45].

At Ch 12 and Ch 13, male children with ASD were associated with significantly smaller oxy-Hb changes than healthy children in the present study. Those channels were localized in the dorsolateral prefrontal cortex, whose hypoactivation was observed in ASD during cognitive control tasks involving inhibition [46], attention [47, 48], and working memory [49, 50]. Two previous studies used 16-channel NIRS during the Stroop task in ASD [30, 31]. However, accurately measuring dorsolateral prefrontal hemodynamic activity is difficult with 16-channel NIRS, which is more suitable for measuring the hemodynamic response of the orbitofrontal and frontopolar cortices. Thus, no significant differences were found between ASD children and typically developing controls in prefrontal hemodynamic activity measured with 16-channel NIRS during the Stroop task. In the current study, we used a 24-channel NIRS system rather than a 16-channel system. The results revealed that male children with ASD exhibited reduced dorsolateral prefrontal hemodynamic responses, measured with 24-channel NIRS during the Stroop color-word task.

In the present study, we used the Stroop color-word task because the prefrontal cortex is reported to be one of the regions most strongly related to Stroop interference [37]. Negoro et al. [26] examined brain activation in 20 healthy children during the Stroop color-word task, measured with NIRS. In that study, oxy-Hb changes indicated specific activation in the prefrontal cortex, and there were positive correlations between the SCWC and age (ages 6–13 years; mean 9.35 ± 2.13 years). The researchers concluded that prefrontal brain activation in healthy children during the Stroop color-word task is similar to that of healthy adults, measured using NIRS [51]. Similar results were obtained in the present study. In both groups, there were positive correlations between SCWC scores and age, and there were no correlations between SCWC scores and FIQ. This result was consistent with previous research on the Stroop that documented that children became progressively faster as they responded verbally to stimuli [52]. These data suggest that the Stroop color-word task used in the present study may be a useful task for children.

Several potential limitations of the present study should be taken into consideration. First, NIRS has several disadvantages compared with other modalities [53]: for example, NIRS enables measurement of Hb concentration changes only as relative values, not as absolute values. We used the Stroop color-word task with a clear baseline task to overcome these potential problems. In addition, we measured Hb concentration changes between the activation task and the baseline task, and performed the task three times to average out the potential effects of incidental changes, and prevent participants from becoming tired. The grand average waveforms of oxy-Hb concentration changes in the ASD group did not indicate a regional cerebral blood flow decrease during the activation task or a difference in blood flow between the baseline and activation tasks. Second, the spatial resolution for detecting hemodynamic responses from the scalp surface using NIRS is lower compared with fMRI, SPECT and PET. However, abnormal prefrontal hemodynamic responses in individuals with ASD are certainly detectible with NIRS. Third, several previous studies have shown that superficial hemodynamic changes, such as skin blood flow, can affect prefrontal NIRS hemoglobin signals [54, 55]. Thus, the present findings could have been influenced by skin blood flow. However, Sato et al. [56] conducted simultaneous NIRS, fMRI, and laser Doppler flowmeter measurements to determine whether prefrontal NIRS hemoglobin signals reflected cortical activity rather than superficial effects. They concluded that NIRS can be used to measure hemodynamic signals originating from prefrontal cortex activation. Fourth, only male children were included in the current study. ASD is more prevalent in males, and gender differences exist in clinical manifestation, cognitive deficits, and brain dysfunctions [32–34, 57, 58]. Thus, our findings may not be generalizable to the female population. Nevertheless, the current finding of abnormal prefrontal hemodynamic responses in male children with ASD is valuable for extending current knowledge. Fifth, the sample size was small, although the 12 male children with ASD were drug-naïve and none had comorbid psychiatric, neurodevelopmental or neurological disorders. However, the required sample size was 11 when we calculated it as α error prob 0.05, power (1-β error prob) 0.8, and effect size 1.3 (effect size in previous studies [23, 26]: 1.3 to 1.6) before the start of this study. In this study, effect size was 1.4 to 1.5. This study is adequately powered (power (1-β error prob): 0.95 to 0.97). Future research with larger sample sizes will be needed to confirm the current findings.

## Conclusion

To our knowledge, this is the first 24-channel NIRS study examining reduced prefrontal hemodynamic responses in male children with ASD during the Stroop color-word task. We found that oxy-Hb changes in 12 drug-naïve male children with ASD were significantly smaller than those in 12 healthy male children in the dorsolateral prefrontal cortex. In addition, 24-channel NIRS systems appears to be a very useful measurement modality for assessing the frontal function of ASD, as it enables non-invasive functional mapping of the cerebral cortex and has much shorter measurement times (about 5 min) compared with other functional brain imaging methodologies.

## Data Availability

The dataset of this study is available from the corresponding author on reasonable request.

## Abbreviations

ASD: autism spectrum disorder
NIRS: near-infrared spectroscopy
oxy-Hb: oxygenated hemoglobin
deoxy-Hb: deoxygenated hemoglobin
PET: positron emission tomography
FIQ: full-scale intelligence quotient
SCWC-1: Stroop color-word task number of correct answers first time
SCWC-2: Stroop color-word task number of correct answers second time
SCWC-3: Stroop color-word task number of correct answers third time
FDR: false discovery rate
fMRI: functional magnetic resonance imaging
SPECT: single-photon emission computed tomography
